# Supplementary material for: Variant abundance estimation for SARS-CoV-2 in wastewater using RNA-Seq quantification
Source: medRxiv. 2021 Sep 2:2021.08.31.21262938. Preprint. [Version 1] doi: 10.1101/2021.08.31.21262938 (PMC8423229; doi:10.1101/2021.08.31.21262938)
Supplement: 1 [file NIHPP2021.08.31.21262938v1-supplement-1.pdf]

medRxiv preprint doi: https://doi.org/10.1101/2021.08.31.21262938; this version posted September 2, 2021. The copyright holder for this preprint (which was not certified by peer review) is the author/funder, who has granted medRxiv a license to display the preprint in perpetuity.
It is made available under a CC-BY 4.0 International license .

Supplementary figures

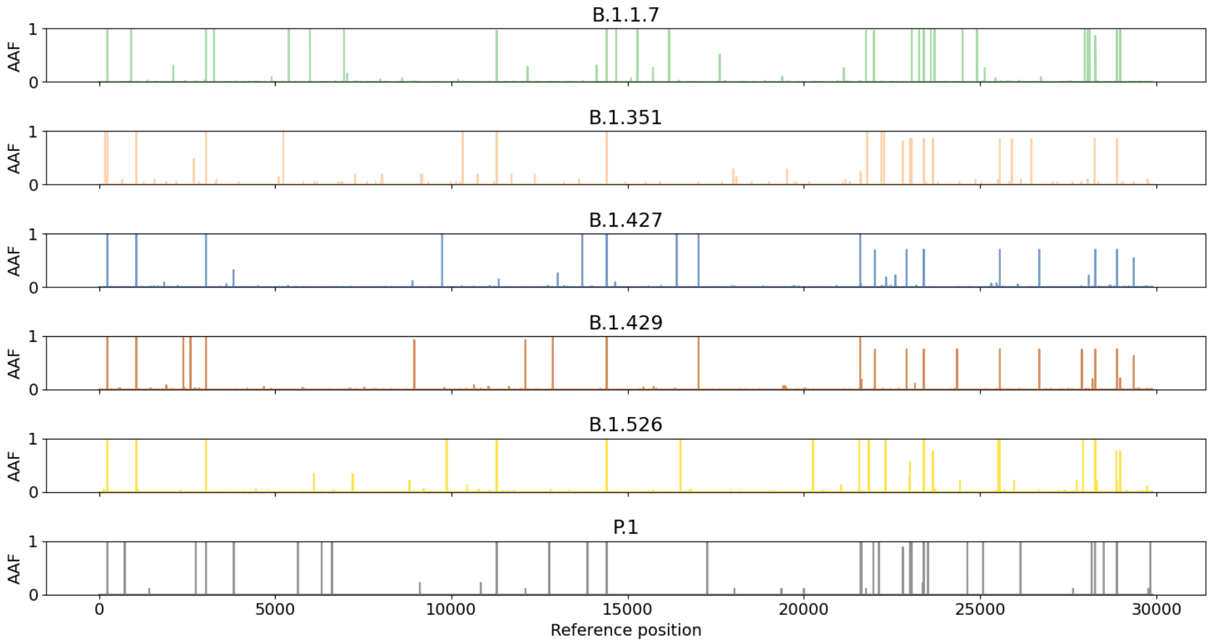

Figure S1: Within-lineage diversity observed in SARS-CoV-2 genomes on GISAID (downloaded 9 March 2021). The horizontal axis shows the position (in base pairs) on the reference genome (accession MN908947.3). The y-axis shows the alternative allele frequency (AAF), i.e. the fraction of genomes with a different nucleotide at a given position than the reference genome. This plot was computed by randomly selecting 1000 genomes of US origin per lineage.

medRxiv preprint doi: https://doi.org/10.1101/2021.08.31.21262938; this version posted September 2, 2021. The copyright holder for this preprint (which was not certified by peer review) is the author/funder, who has granted medRxiv a license to display the preprint in perpetuity.
It is made available under a CC-BY 4.0 International license .

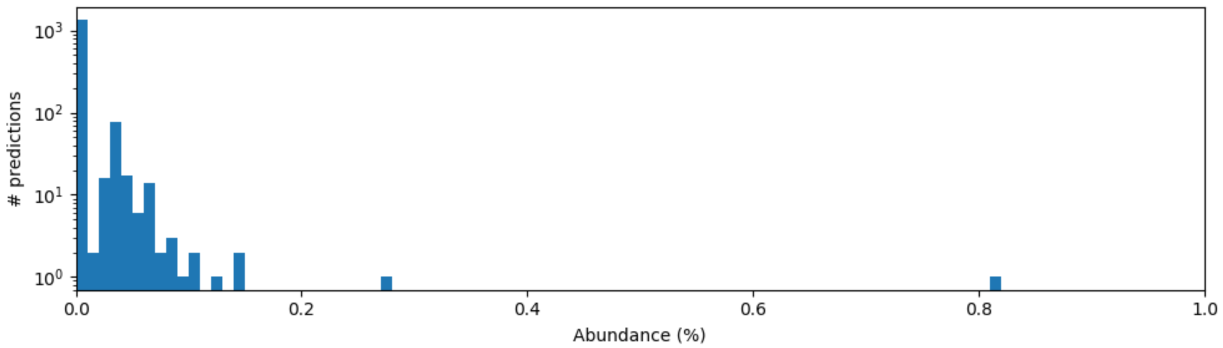

Figure S2. Histogram of raw abundances predicted by kallisto on a simulated dataset consisting of 100% B.1.1.7. The majority of false positives (background noise) can be filtered out by applying a minimal abundance threshold of 0.1%. True predictions occur at higher abundances (beyond the x-axis limit of 1.0%).

medRxiv preprint doi: https://doi.org/10.1101/2021.08.31.21262938; this version posted September 2, 2021. The copyright holder for this preprint (which was not certified by peer review) is the author/funder, who has granted medRxiv a license to display the preprint in perpetuity.
It is made available under a CC-BY 4.0 International license .

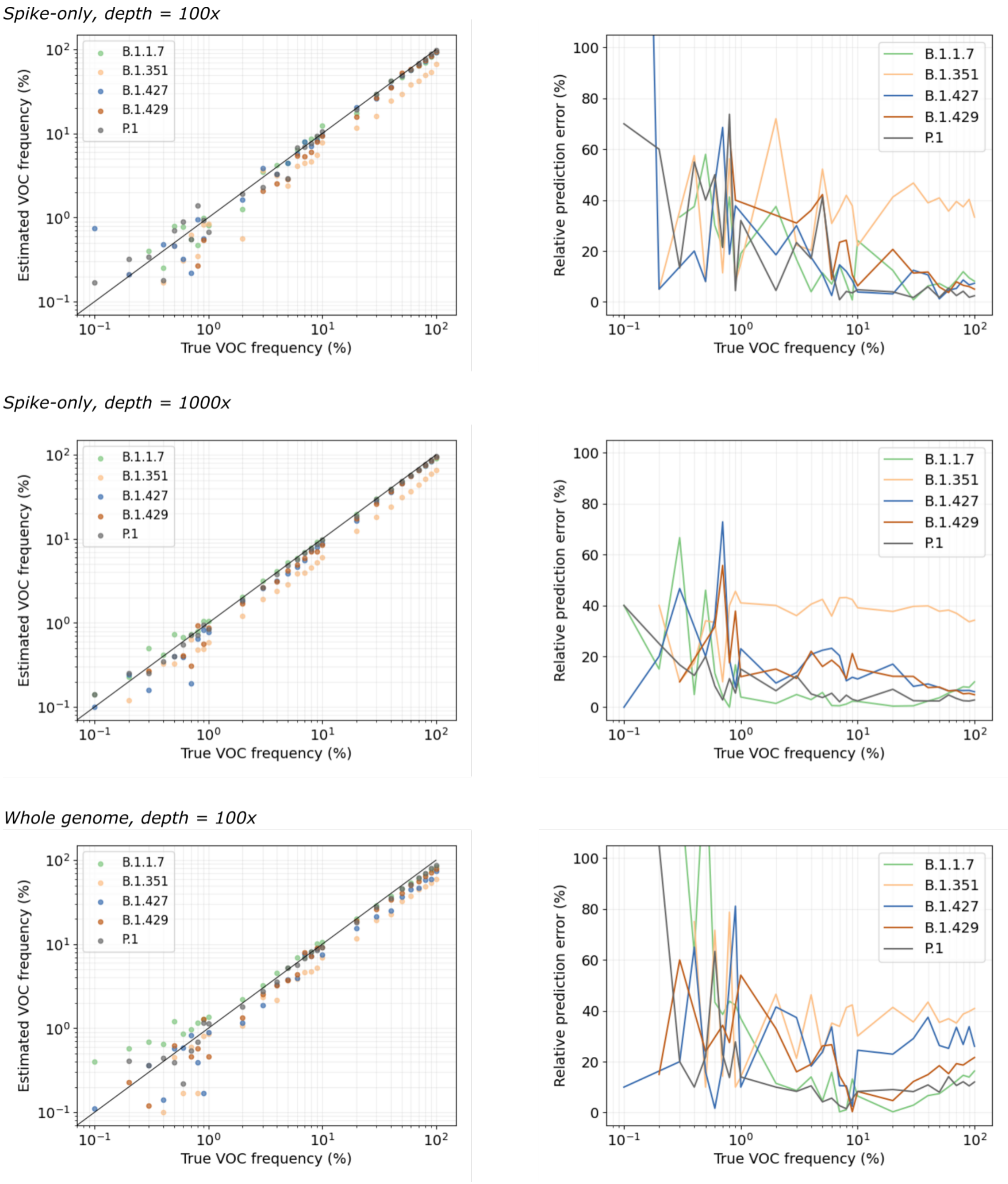

Figure S3: Additional benchmarking results

medRxiv preprint doi: https://doi.org/10.1101/2021.08.31.21262938; this version posted September 2, 2021. The copyright holder for this preprint
(which was not certified by peer review) is the author/funder, who has granted medRxiv a license to display the preprint in perpetuity.
It is made available under a CC-BY 4.0 International license .

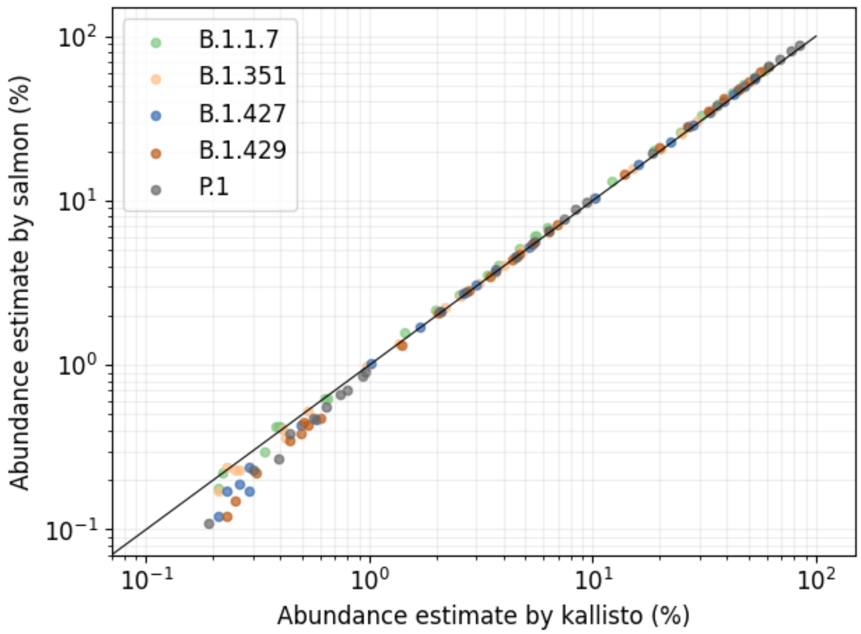

Figure S4: salmon versus kallisto abundance estimates per variant.

medRxiv preprint doi: https://doi.org/10.1101/2021.08.31.21262938; this version posted September 2, 2021. The copyright holder for this preprint (which was not certified by peer review) is the author/funder, who has granted medRxiv a license to display the preprint in perpetuity.
It is made available under a CC-BY 4.0 International license .

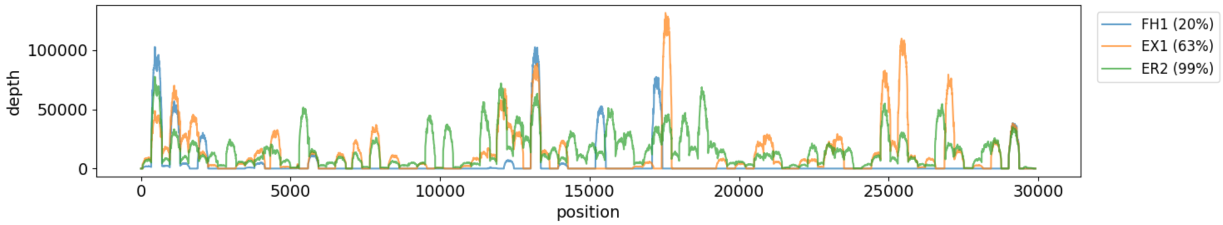

Figure S5: Sequencing depth from wastewater samples is highly uneven between amplicons. This figure shows sequencing depth along the genome for three samples collected in New Haven, CT. The first sample (FH1) has low genome coverage (20%), with very few amplicons reaching high sequencing depth. The second sample (EX1) has moderate genome coverage (63%), with roughly half of the amplicons reaching high sequencing depth. The third sample (ER2) has high genome coverage (99%), with nearly all amplicons reaching high sequencing depth.

medRxiv preprint doi: https://doi.org/10.1101/2021.08.31.21262938; this version posted September 2, 2021. The copyright holder for this preprint (which was not certified by peer review) is the author/funder, who has granted medRxiv a license to display the preprint in perpetuity.
It is made available under a CC-BY 4.0 International license .

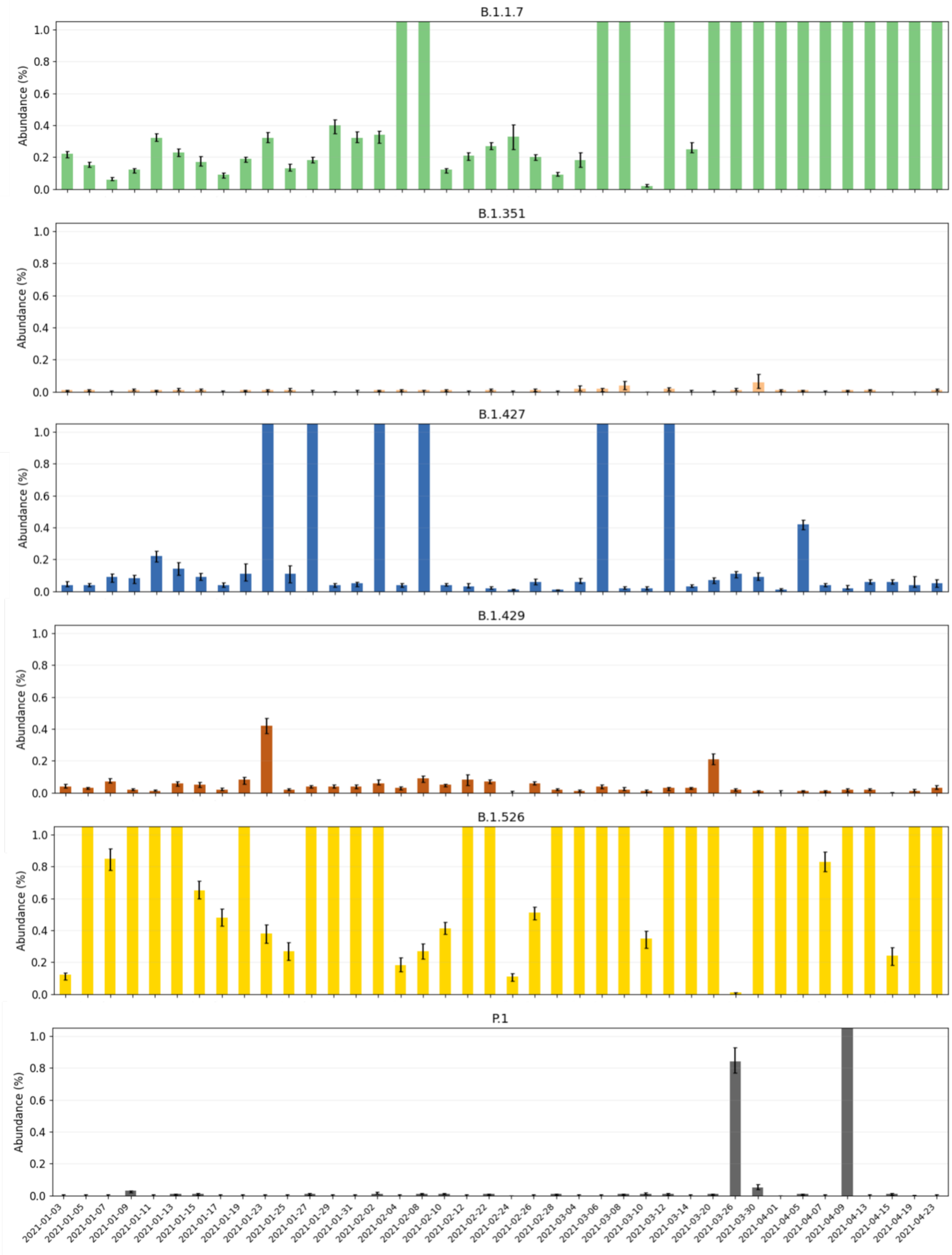

Figure S6: Raw predictions per variant with confidence intervals based on bootstrap analysis for New Haven samples. Note that in all subplots the y-axis is capped at 1% for improved readability.

medRxiv preprint doi: https://doi.org/10.1101/2021.08.31.21262938; this version posted September 2, 2021. The copyright holder for this preprint
(which was not certified by peer review) is the author/funder, who has granted medRxiv a license to display the preprint in perpetuity.
It is made available under a CC-BY 4.0 International license .

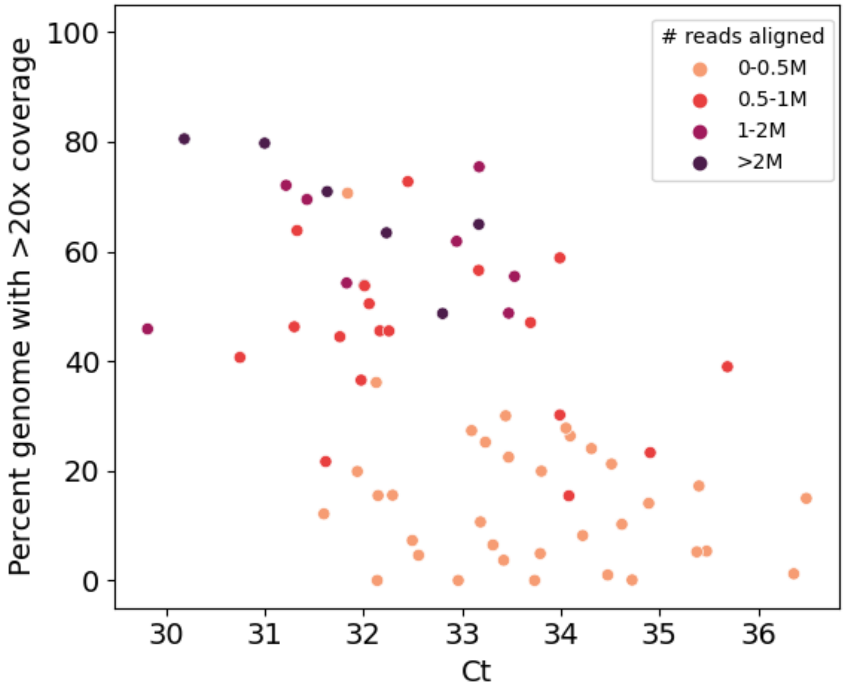

Figure S7: Percent genome coverage versus Ct values for samples across the US

medRxiv preprint doi: https://doi.org/10.1101/2021.08.31.21262938; this version posted September 2, 2021. The copyright holder for this preprint (which was not certified by peer review) is the author/funder, who has granted medRxiv a license to display the preprint in perpetuity.
It is made available under a CC-BY 4.0 International license .

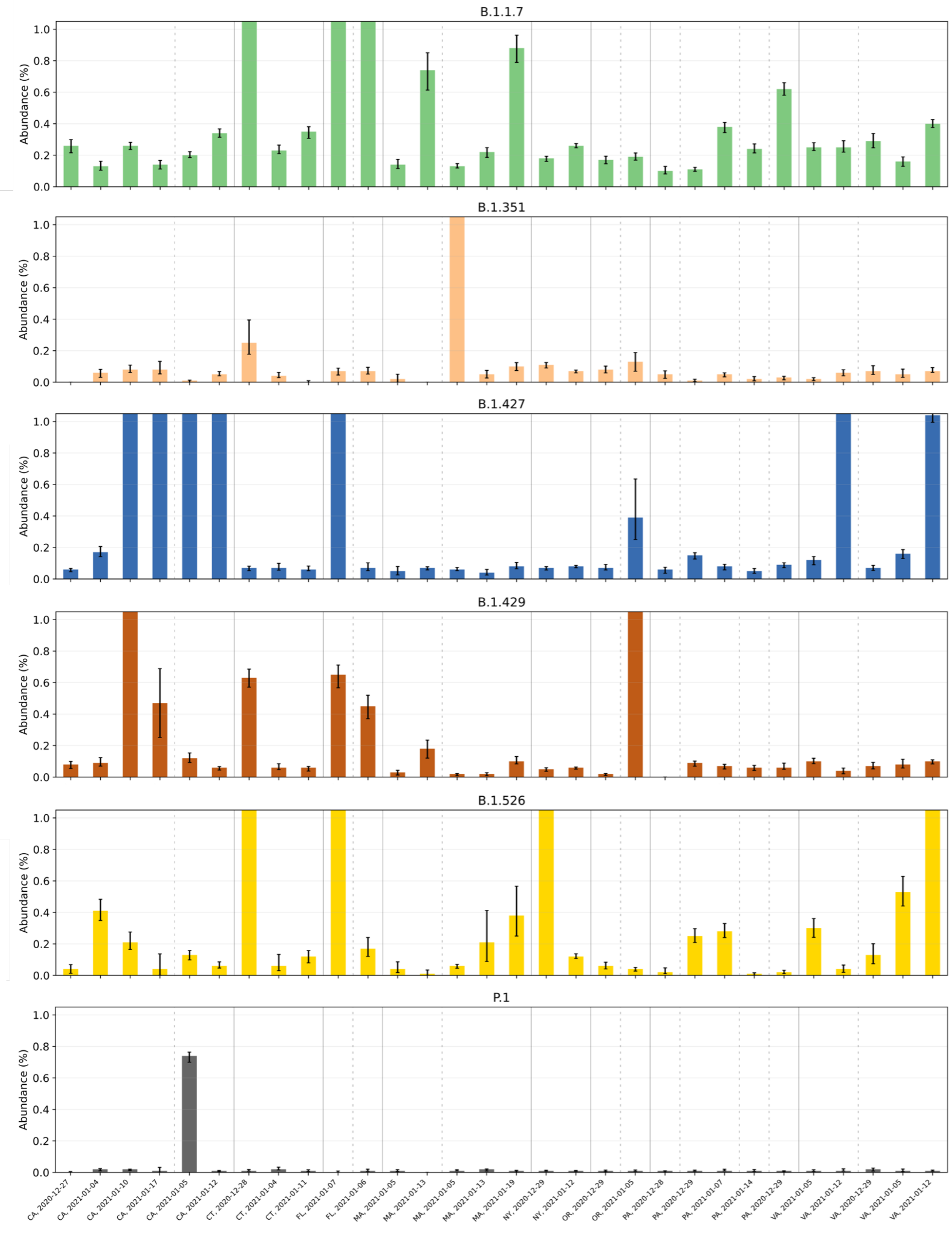

Figure S8: Raw predictions per variant with confidence intervals based on bootstrap analysis for samples across the US. Note that in all subplots the y-axis is capped at 1% for improved readability.
